# Supplementary material for: Conserved DNA motifs in the type II-A CRISPR leader region
Source: PeerJ. 2017 Apr 4;5:e3161. doi: 10.7717/peerj.3161 (PMC5382924; doi:10.7717/peerj.3161)
Supplement: Supplemental Information 1 — Supplementary Figure S1 (A) Sequence alignment of the last 20 nucleotides of the leader and the first repeat of all 87 Group 1 loci. Height of the letters in the WebLogo indicates the degree of conservation at specific nucleotide locations. (B) Sequence alignment of the last 20 nucleotides of the leader and the first repeat of all 55 Group 2 loci. (C) Sequence alignment of the last 20 nucleotides of the leader and the first repeat of all 25 Group 3 loci. Supplementary Figure S2 Phylogenetic tree generated from the sequence alignment of the first repeat from all 167 type II-A loci. Groups based on the segregation of the Cas1 tree are shown in cyan (Group 1), red (Group 2), and yellow (Group 3). Supplementary Figure S3 Phylogenetic tree generated from the sequence alignment of the Cas1 from all 167 type II-A loci. Groups are shown in cyan (Group 1), red (Group 2), and yellow (Group 3). Supplementary Figure S4 (A) Phylogenetic tree generated from the sequence alignment of Cas9 from all 167 loci. Groups based on the segregation of the Cas1 tree are shown in cyan (Group 1), red (Group 2), and yellow (Group 3). (B) Phylogenetic tree generated from the sequence alignment of Cas2. Supplementary Figure S5 Phylogenetic tree generated from the sequence alignment of Csn2. Groups based on the segregation of the Cas1 tree are shown in cyan (Group 1), red (Group 2), and yellow (Group 3). Supplementary Table S1 List of all 167 species used in this study including the strain name, abbreviation used, and the nucleotide accession number. Supplementary Table S2 Repeats that showed differences in length and sequence based on our in-house script and the CRISPRdb. Supplementary Table S3 Conservation of protein lengths shown by averages of protein lengths within particular branches. Groups are based on the Csn2 tree, 1a referring to the ancestral branch of Group 1 and 1b referring to the other. Branches in all the trees are not in the same position, but the same segregation is kept across [file peerj-05-3161-s001.pdf]

Figure S1

A

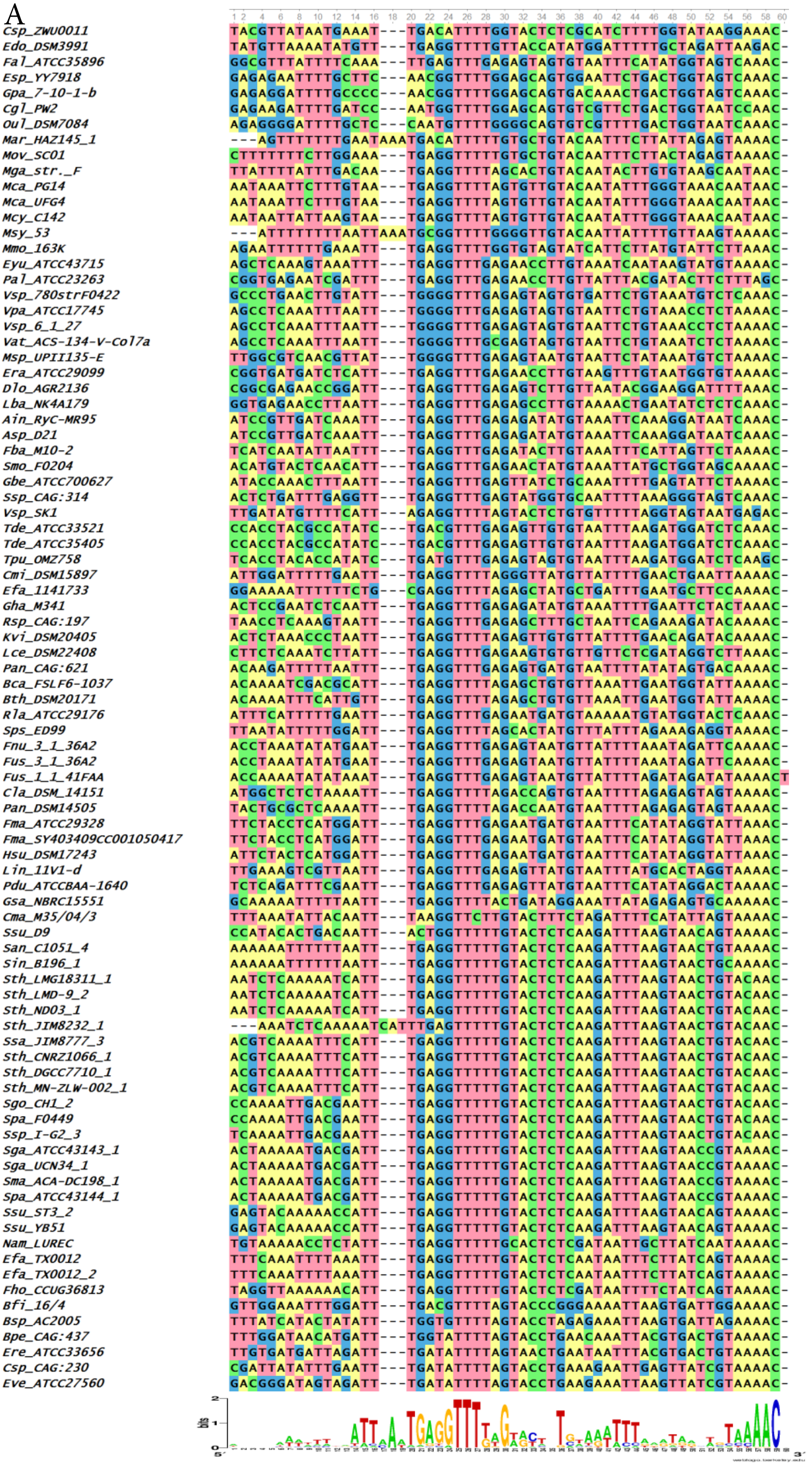
Csp_ZWU0011
Edo_DSM3991
Fal_ATCC35896
Esp_YY7918
Gpa_7-10-1-b
Cgl_PW2
Oul_DSM7084
Mar_HAZ145_1
Mov_SC01
Mga_str._F
Mca_PG14
Mca_UFG4
Mcy_C142
Msy_53
Mmo_163K
Eyu_ATCC43715
Pal_ATCC23263
Vsp_780strF0422
Vpa_ATCC17745
Vsp_6_1_27
Vat_ACS-134-V-Col7a
Msp_UPII135-E
Era_ATCC29099
Dlo_AGR2136
Lba_NK4A179
Ain_RyC-MR95
Asp_D21
Fba_M10-2
Smo_F0204
Gbe_ATCC700627
Ssp_CAG:314
Vsp_SK1
Tde_ATCC33521
Tde_ATCC35405
Tpu_OMZ758
Cmi_DSM15897
Efa_1141733
Gha_M341
Rsp_CAG:197
Kvi_DSM20405
Lce_DSM22408
Pan_CAG:621
Bca_FSLF6-1037
Bth_DSM20171
Rla_ATCC29176
Sps_ED99
Fnu_3_1_36A2
Fus_3_1_36A2
Fus_1_1_41FAA
Cla_DSM_14151
Pan_DSM14505
Fma_ATCC29328
Fma_SY403409CC001050417
Hsu_DSM17243
Lin_11V1-d
Pdu_ATCCBAA-1640
Gsa_NBRC15551
Cma_M35/04/3
Ssu_D9
San_C1051_4
Sin_B196_1
Sth_LMG18311_1
Sth_LMD-9_2
Sth_ND03_1
Sth_JIM8232_1
Ssa_JIM8777_3
Sth_CNRZ1066_1
Sth_DGCC7710_1
Sth_MN-ZLW-002_1
Sgo_CH1_2
Spa_F0449
Ssp_I-G2_3
Sga_ATCC43143_1
Sga_UCN34_1
Sma_ACA-DC198_1
Spa_ATCC43144_1
Ssu_ST3_2
Ssu_YB51
Nam_LUREC
Efa_TX0012
Efa_TX0012_2
Fho_CCUG36813
Bfi_16/4
Bsp_AC2005
Bpe_CAG:437
Ere_ATCC33656
Csp_CAG:230
Eve_ATCC27560
bits
5'
3'

B

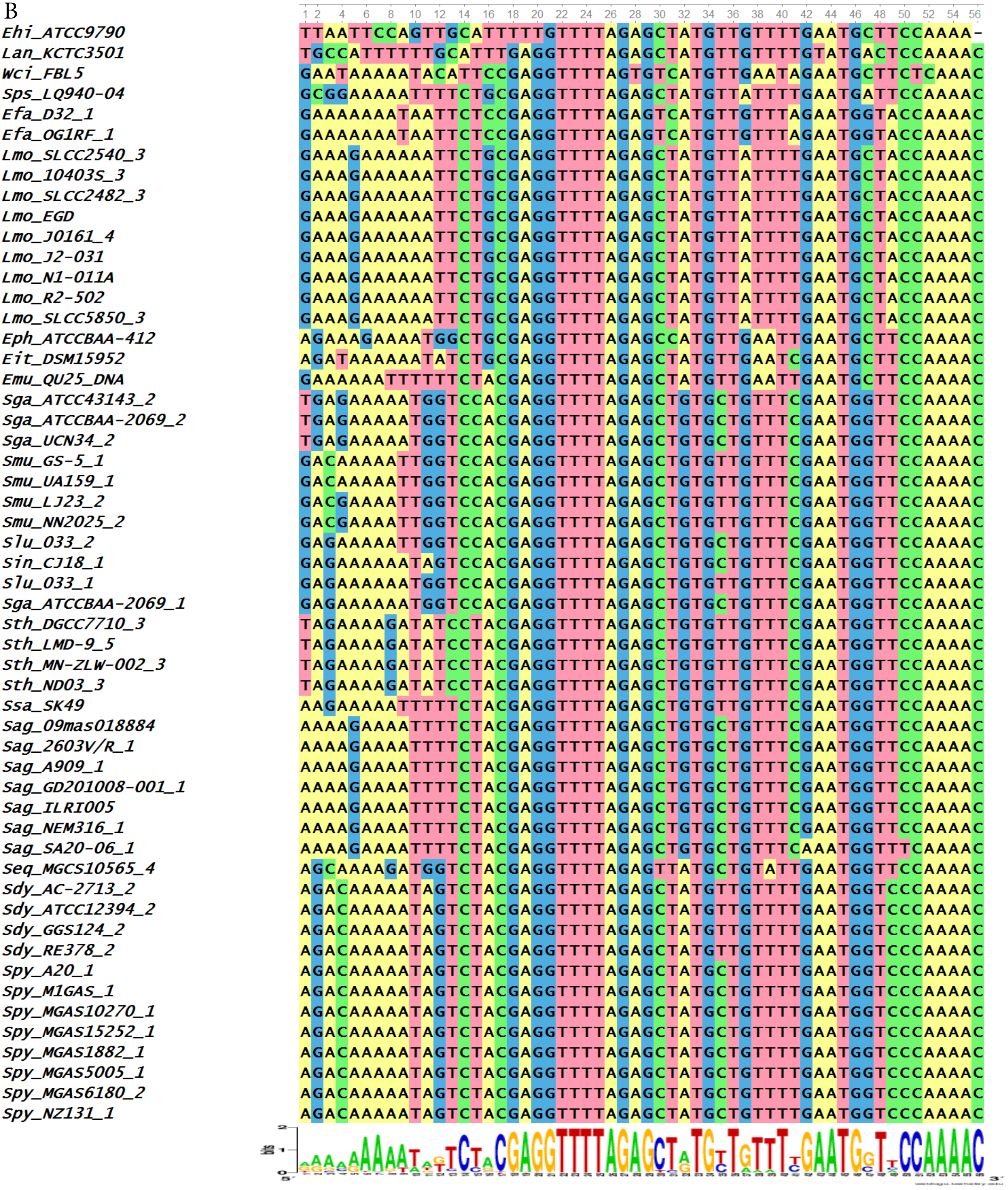
Ehi_ATCC9790 TTAATTCCAGTTGCATTTTTGTTTTAGAGCTATGTTGTTTTGAATGCTTCCAAAA-
Lan_KCTC3501 TGCCATTTTTTGCATTTGAGGTTTTAGAGCTATGTTGTTTTGTATGACTCCAAAAC
Wci_FBL5 GAATAAAAATACATTCCGAGGTTTTAGTGTCATGTTGAATAGAATGCTTCTCAAAC
Sps_LQ940-04 GCGGAAAAATTTTCTGCGAGGTTTTAGAGCTATGTTATTTTGAATGATTCCAAAAC
Efa_D32_1 GAAAAAAATAATTCTCCGAGGTTTTAGAGTCATGTTGTTTAGAATGGTACCAAAAC
Efa_OG1RF_1 GAAAAAAATAATTCTCCGAGGTTTTAGAGTCATGTTGTTTAGAATGGTACCAAAAC
Lmo_SLCC2540_3 GAAAGAAAAAATTCTGCGAGGTTTTAGAGCTATGTTATTTTGAATGCTACCAAAAC
Lmo_10403S_3 GAAAGAAAAAATTCTGCGAGGTTTTAGAGCTATGTTATTTTGAATGCTACCAAAAC
Lmo_SLCC2482_3 GAAAGAAAAAATTCTGCGAGGTTTTAGAGCTATGTTATTTTGAATGCTACCAAAAC
Lmo_EGD GAAAGAAAAAATTCTGCGAGGTTTTAGAGCTATGTTATTTTGAATGCTACCAAAAC
Lmo_J0161_4 GAAAGAAAAAATTCTGCGAGGTTTTAGAGCTATGTTATTTTGAATGCTACCAAAAC
Lmo_J2-031 GAAAGAAAAAATTCTGCGAGGTTTTAGAGCTATGTTATTTTGAATGCTACCAAAAC
Lmo_N1-011A GAAAGAAAAAATTCTGCGAGGTTTTAGAGCTATGTTATTTTGAATGCTACCAAAAC
Lmo_R2-502 GAAAGAAAAAATTCTGCGAGGTTTTAGAGCTATGTTATTTTGAATGCTACCAAAAC
Lmo_SLCC5850_3 GAAAGAAAAAATTCTGCGAGGTTTTAGAGCTATGTTATTTTGAATGCTACCAAAAC
Eph_ATCCBAA-412 AGAAAGAAAATGGCTGCGAGGTTTTAGAGCCATGTTGAATTGAATGCTTCCAAAAC
Eit_DSM15952 AGATAAAAAATATCTGCGAGGTTTTAGAGCTATGTTGAATCGAATGCTTCCAAAAC
Emu_QU25_DNA GAAAAAATTTTTTCTACGAGGTTTTAGAGCTATGTTGAATTGAATGCTTCCAAAAC
Sga_ATCC43143_2 TGAGAAAAATGGTCCACGAGGTTTTAGAGCTGTGCTGTTTCGAATGGTTCCAAAAC
Sga_ATCCBAA-2069_2 TGAGAAAAATGGTCCACGAGGTTTTAGAGCTGTGCTGTTTCGAATGGTTCCAAAAC
Sga_UCN34_2 TGAGAAAAATGGTCCACGAGGTTTTAGAGCTGTGCTGTTTCGAATGGTTCCAAAAC
Smu_GS-5_1 GACAAAAATTGGTCCACGAGGTTTTAGAGCTGTGTTGTTTCGAATGGTTCCAAAAC
Smu_UA159_1 GACAAAAATTGGTCCACGAGGTTTTAGAGCTGTGTTGTTTCGAATGGTTCCAAAAC
Smu_LJ23_2 GACGAAAATTGGTCCACGAGGTTTTAGAGCTGTGTTGTTTCGAATGGTTCCAAAAC
Smu_NN2025_2 GACGAAAATTGGTCCACGAGGTTTTAGAGCTGTGTTGTTTCGAATGGTTCCAAAAC
Slu_033_2 GAGAAAAATTGGTCCACGAGGTTTTAGAGCTGTGCTGTTTCGAATGGTTCCAAAAC
Sin_CJ18_1 GAGAAAAAATAGTCCACGAGGTTTTAGAGCTGTGCTGTTTCGAATGGTTCCAAAAC
Slu_033_1 GAGAAAAAATGGTCCACGAGGTTTTAGAGCTGTGTTGTTTCGAATGGTTCCAAAAC
Sga_ATCCBAA-2069_1 GAGAAAAAATGGTCCACGAGGTTTTAGAGCTGTGCTGTTTCGAATGGTTCCAAAAC
Sth_DGCC7710_3 TAGAAAAGATATCCTACGAGGTTTTAGAGCTGTGTTGTTTCGAATGGTTCCAAAAC
Sth_LMD-9_5 TAGAAAAGATATCCTACGAGGTTTTAGAGCTGTGTTGTTTCGAATGGTTCCAAAAC
Sth_MN-ZLW-002_3 TAGAAAAGATATCCTACGAGGTTTTAGAGCTGTGTTGTTTCGAATGGTTCCAAAAC
Sth_ND03_3 TAGAAAAGATATCCTACGAGGTTTTAGAGCTGTGTTGTTTCGAATGGTTCCAAAAC
Ssa_SK49 AAGAAAAATTTTTCTACGAGGTTTTAGAGCTGTGTTGTTTCGAATGGTTCCAAAAC
Sag_09mas018884 AAAAGAAAATTTTCTACGAGGTTTTAGAGCTGTGCTGTTTCGAATGGTTCCAAAAC
Sag_2603V/R_1 AAAAGAAAATTTTCTACGAGGTTTTAGAGCTGTGCTGTTTCGAATGGTTCCAAAAC
Sag_A909_1 AAAAGAAAATTTTCTACGAGGTTTTAGAGCTGTGCTGTTTCGAATGGTTCCAAAAC
Sag_GD201008-001_1 AAAAGAAAATTTTCTACGAGGTTTTAGAGCTGTGCTGTTTCGAATGGTTCCAAAAC
Sag_ILRI005 AAAAGAAAATTTTCTACGAGGTTTTAGAGCTGTGCTGTTTCGAATGGTTCCAAAAC
Sag_NEM316_1 AAAAGAAAATTTTCTACGAGGTTTTAGAGCTGTGCTGTTTCGAATGGTTCCAAAAC
Sag_SA20-06_1 AAAAGAAAATTTTCTACGAGGTTTTAGAGCTGTGCTGTTTCAAATGGTTTCAAAAC
Seq_MGCS10565_4 AGCAAAAGATGGTCTACGAGGTTTTAGAGTTATGCTGTATTGAATGGTTCCAAAAC
Sdy_AC-2713_2 AGACAAAAATAGTCTACGAGGTTTTAGAGCTATGTTGTTTTGAATGGTCCCAAAAC
Sdy_ATCC12394_2 AGACAAAAATAGTCTACGAGGTTTTAGAGCTATGTTGTTTTGAATGGTCCCAAAAC
Sdy_GGS124_2 AGACAAAAATAGTCTACGAGGTTTTAGAGCTATGTTGTTTTGAATGGTCCCAAAAC
Sdy_RE378_2 AGACAAAAATAGTCTACGAGGTTTTAGAGCTATGTTGTTTTGAATGGTCCCAAAAC
Spy_A20_1 AGACAAAAATAGTCTACGAGGTTTTAGAGCTATGCTGTTTTGAATGGTCCCAAAAC
Spy_M1GAS_1 AGACAAAAATAGTCTACGAGGTTTTAGAGCTATGCTGTTTTGAATGGTCCCAAAAC
Spy_MGAS10270_1 AGACAAAAATAGTCTACGAGGTTTTAGAGCTATGCTGTTTTGAATGGTCCCAAAAC
Spy_MGAS15252_1 AGACAAAAATAGTCTACGAGGTTTTAGAGCTATGCTGTTTTGAATGGTCCCAAAAC
Spy_MGAS1882_1 AGACAAAAATAGTCTACGAGGTTTTAGAGCTATGCTGTTTTGAATGGTCCCAAAAC
Spy_MGAS5005_1 AGACAAAAATAGTCTACGAGGTTTTAGAGCTATGCTGTTTTGAATGGTCCCAAAAC
Spy_MGAS6180_2 AGACAAAAATAGTCTACGAGGTTTTAGAGCTATGCTGTTTTGAATGGTCCCAAAAC
Spy_NZ131_1 AGACAAAAATAGTCTACGAGGTTTTAGAGCTATGCTGTTTTGAATGGTCCCAAAAC
bits
5′
3′

C

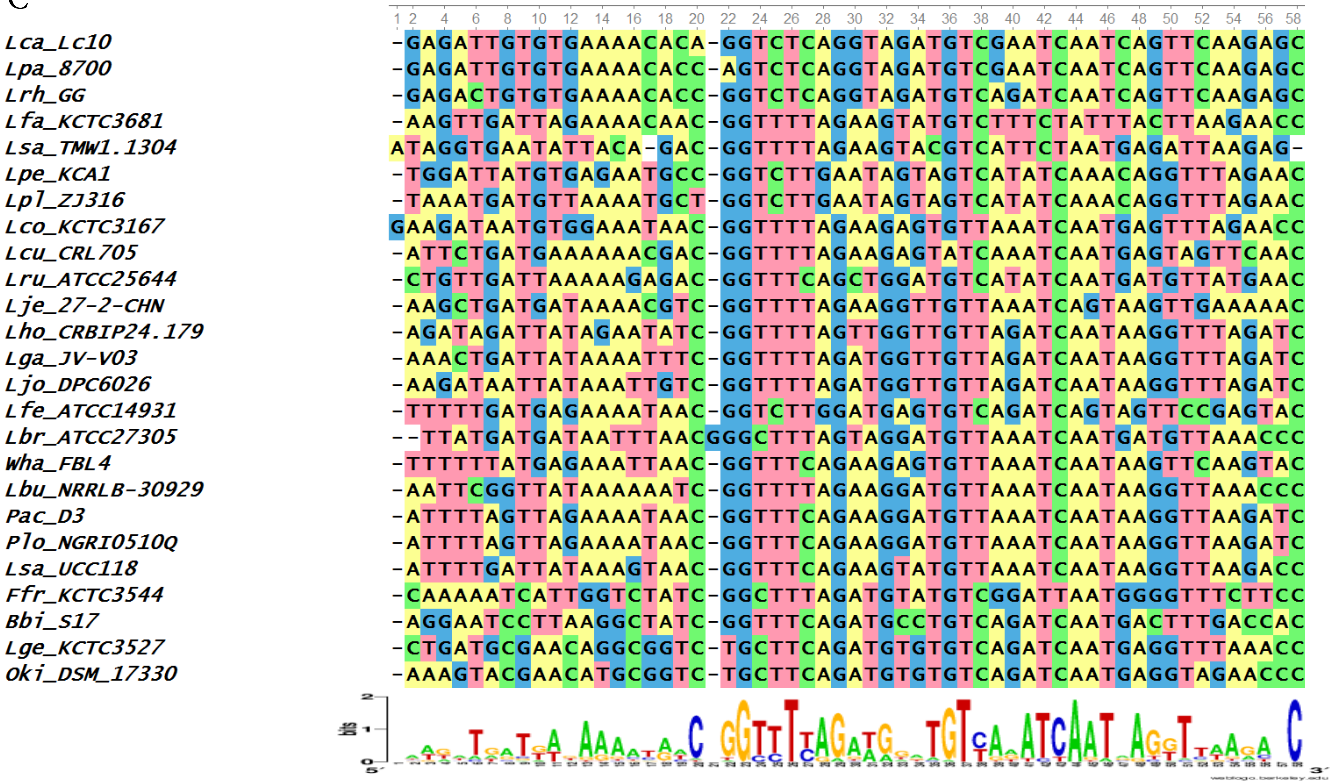
1 2 4 6 8 10 12 14 16 18 20 22 24 26 28 30 32 34 36 38 40 42 44 46 48 50 52 54 56 58
Lca_Lc10 -GAGATTGTGTGAAAACACA-GGTCTCAGGTAGATGTCGAATCAATCAGTTCAAGAGC
Lpa_8700 -GAGATTGTGTGAAAACACC-AGTCTCAGGTAGATGTCGAATCAATCAGTTCAAGAGC
Lrh_GG -GAGACTGTGTGAAAACACC-GGTCTCAGGTAGATGTCAGATCAATCAGTTCAAGAGC
Lfa_KCTC3681 -AAGTTGATTAGAAAACAAC-GGTTTTAGAAGTATGTCTTTCTATTTACTTAAGAACC
Lsa_TMW1.1304 ATAGGTGAATATTACA-GAC-GGTTTTAGAAGTACGTCATTCTAATGAGATTAAGAG-
Lpe_KCA1 -TGGATTATGTGAGAATGCC-GGTCTTGAATAGTAGTCATATCAAACAGGTTTAGAAC
Lpl_ZJ316 -TAAATGATGTTAAAATGCT-GGTCTTGAATAGTAGTCATATCAAACAGGTTTAGAAC
Lco_KCTC3167 GAAGATAATGTGGAAATAAC-GGTTTTAGAAGAGTGTTAAATCAATGAGTTTAGAACC
Lcu_CRL705 -ATTCTGATGAAAAAACGAC-GGTTTTAGAAGAGTATCAAATCAATGAGTAGTTCAAC
Lru_ATCC25644 -CTGTTGATTAAAAAGAGAC-GGTTTCAGCTGGATGTCATATCAATGATGTTATGAAC
Lje_27-2-CHN -AAGCTGATGATAAAACGTC-GGTTTTAGAAGGTTGTTAAATCAGTAAGTTGAAAAAC
Lho_CRBIP24.179 -AGATAGATTATAGAATATC-GGTTTTAGTTGGTTGTTAGATCAATAAGGTTTAGATC
Lga_JV-V03 -AAACTGATTATAAAATTTC-GGTTTTAGATGGTTGTTAGATCAATAAGGTTTAGATC
Ljo_DPC6026 -AAGATAATTATAAATTGTC-GGTTTTAGATGGTTGTTAGATCAATAAGGTTTAGATC
Lfe_ATCC14931 -TTTTTGATGAGAAAATAAC-GGTCTTGGATGAGTGTCAGATCAGTAGTTCCGAGTAC
Lbr_ATCC27305 --TTATGATGATAATTTAACGGGCTTTAGTAGGATGTTAAATCAATGATGTTAAACCC
Wha_FBL4 -TTTTTTATGAGAAATTAAC-GGTTTCAGAAGAGTGTTAAATCAATAAGTTCAAGTAC
Lbu_NRRLB-30929 -AATTCGGTTATAAAAAATC-GGTTTTAGAAGGATGTTAAATCAATAAGGTTAAACCC
Pac_D3 -ATTTTAGTTAGAAAATAAC-GGTTTCAGAAGGATGTTAAATCAATAAGGTTAAGATC
Plo_NGRI0510Q -ATTTTAGTTAGAAAATAAC-GGTTTCAGAAGGATGTTAAATCAATAAGGTTAAGATC
Lsa_UCC118 -ATTTTGATTATAAAGTAAC-GGTTTCAGAAGTATGTTAAATCAATAAGGTTAAGACC
Ffr_KCTC3544 -CAAAAATCATTGGTCTATC-GGCTTTAGATGTATGTCGGATTAATGGGGTTTCTTCC
Bbi_S17 -AGGAATCCTTAAGGCTATC-GGTTTCAGATGCCTGTCAGATCAATGACTTTGACCAC
Lge_KCTC3527 -CTGATGCGAACAGGCGGTC-TGCTTCAGATGTGTGTCAGATCAATGAGGTTTAAACC
Oki_DSM_17330 -AAAGTACGAACATGCGGTC-TGCTTCAGATGTGTGTCAGATCAATGAGGTAGAACCC
bits
2
1
0
5′
3′
weblogo.berkeley.edu

Figure S2

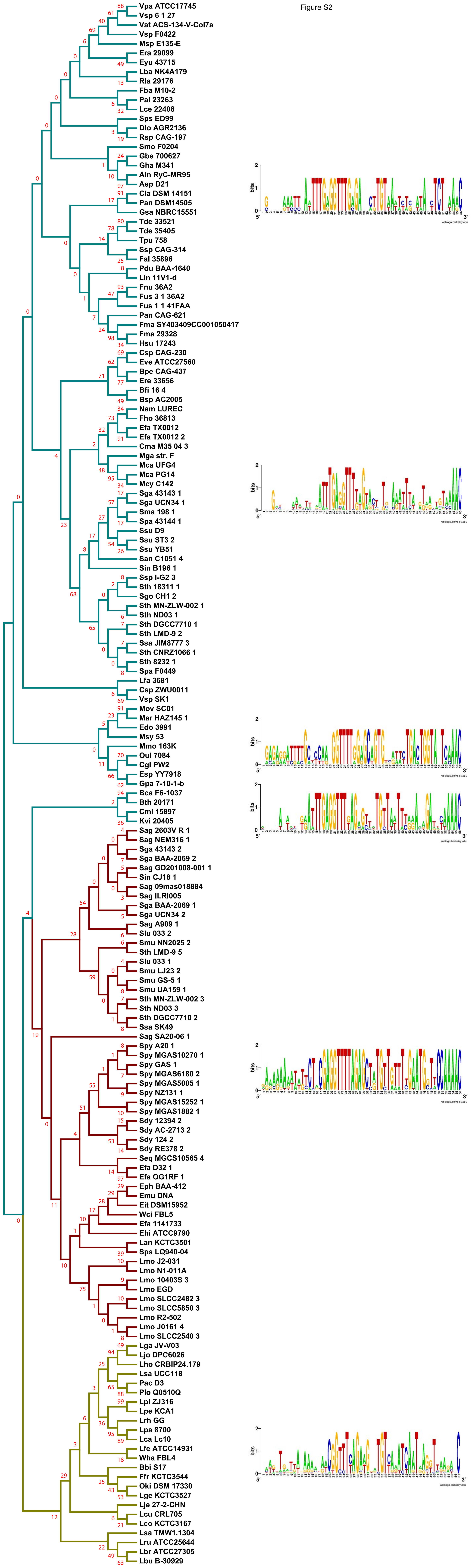

Vpa ATCC17745
Vsp 6 1 27
Vat ACS-134-V-Col7a
Vsp F0422
Msp E135-E
Era 29099
Eyu 43715
Lba NK4A179
Rla 29176
Fba M10-2
Pal 23263
Lce 22408
Sps ED99
Dlo AGR2136
Rsp CAG-197
Smo F0204
Gbe 700627
Gha M341
Ain RyC-MR95
Asp D21
Cla DSM 14151
Pan DSM14505
Gsa NBRC15551
Tde 33521
Tde 35405
Tpu 758
Ssp CAG-314
Fal 35896
Pdu BAA-1640
Lin 11V1-d
Fnu 36A2
Fus 3 1 36A2
Fus 1 1 41FAA
Pan CAG-621
Fma SY403409CC001050417
Fma 29328
Hsu 17243
Csp CAG-230
Eve ATCC27560
Bpe CAG-437
Ere 33656
Bfi 16 4
Bsp AC2005
Nam LUREC
Fho 36813
Efa TX0012
Efa TX0012 2
Cma M35 04 3
Mga str. F
Mca UFG4
Mca PG14
Mcy C142
Sga 43143 1
Sga UCN34 1
Sma 198 1
Spa 43144 1
Ssu D9
Ssu ST3 2
Ssu YB51
San C1051 4
Sin B196 1
Ssp I-G2 3
Sth 18311 1
Sgo CH1 2
Sth MN-ZLW-002 1
Sth ND03 1
Sth DGCC7710 1
Sth LMD-9 2
Ssa JIM8777 3
Sth CNRZ1066 1
Sth 8232 1
Spa F0449
Lfa 3681
Csp ZWU0011
Vsp SK1
Mov SC01
Mar HAZ145 1
Edo 3991
Msy 53
Mmo 163K
Oul 7084
Cgl PW2
Esp YY7918
Gpa 7-10-1-b
Bca F6-1037
Bth 20171
Cmi 15897
Kvi 20405
Sag 2603V R 1
Sag NEM316 1
Sga 43143 2
Sga BAA-2069 2
Sag GD201008-001 1
Sin CJ18 1
Sag 09mas018884
Sag ILRI005
Sga BAA-2069 1
Sga UCN34 2
Sag A909 1
Slu 033 2
Smu NN2025 2
Sth LMD-9 5
Slu 033 1
Smu LJ23 2
Smu GS-5 1
Smu UA159 1
Sth MN-ZLW-002 3
Sth ND03 3
Sth DGCC7710 2
Ssa SK49
Sag SA20-06 1
Spy A20 1
Spy MGAS10270 1
Spy GAS 1
Spy MGAS6180 2
Spy MGAS5005 1
Spy NZ131 1
Spy MGAS15252 1
Spy MGAS1882 1
Sdy 12394 2
Sdy AC-2713 2
Sdy 124 2
Sdy RE378 2
Seq MGCS10565 4
Efa D32 1
Efa OG1RF 1
Eph BAA-412
Emu DNA
Eit DSM15952
Wci FBL5
Efa 1141733
Ehi ATCC9790
Lan KCTC3501
Sps LQ940-04
Lmo J2-031
Lmo N1-011A
Lmo 10403S 3
Lmo EGD
Lmo SLCC2482 3
Lmo SLCC5850 3
Lmo R2-502
Lmo J0161 4
Lmo SLCC2540 3
Lga JV-V03
Ljo DPC6026
Lho CRBIP24.179
Lsa UCC118
Pac D3
Plo Q0510Q
Lpl ZJ316
Lpe KCA1
Lrh GG
Lpa 8700
Lca Lc10
Lfe ATCC14931
Wha FBL4
Bbi S17
Ffr KCTC3544
Oki DSM 17330
Lge KCTC3527
Lje 27-2-CHN
Lcu CRL705
Lco KCTC3167
Lsa TMW1.1304
Lru ATCC25644
Lbr ATCC27305
Lbu B-30929

Figure S3

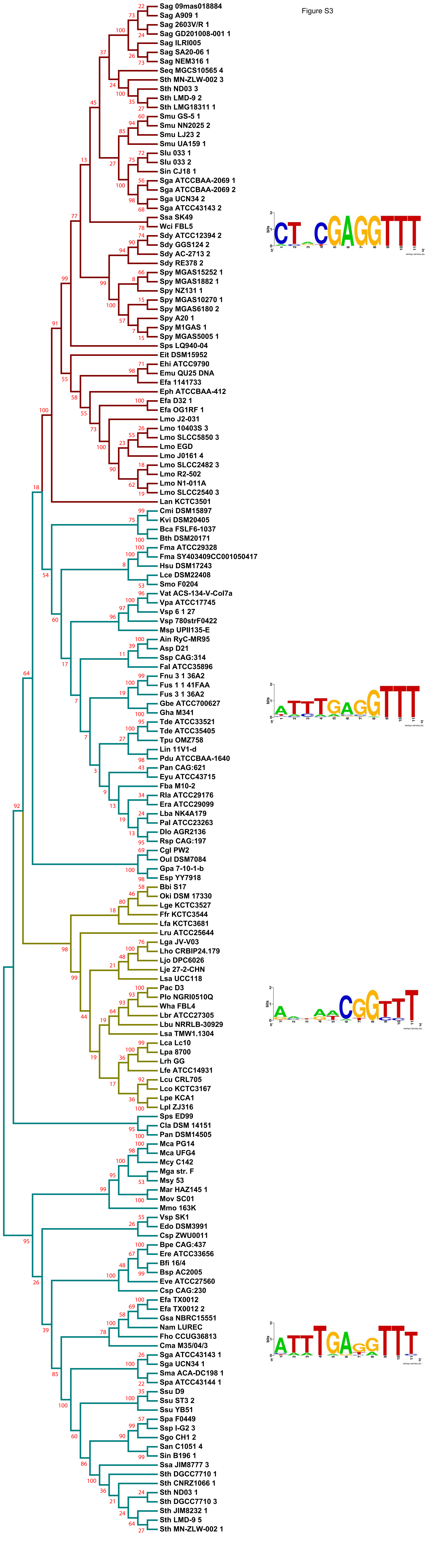
Sag 09mas018884
Sag A909 1
Sag 2603V/R 1
Sag GD201008-001 1
Sag ILRI005
Sag SA20-06 1
Sag NEM316 1
Seq MGCS10565 4
Sth MN-ZLW-002 3
Sth ND03 3
Sth LMD-9 2
Sth LMG18311 1
Smu GS-5 1
Smu NN2025 2
Smu LJ23 2
Smu UA159 1
Slu 033 1
Slu 033 2
Sin CJ18 1
Sga ATCCBAA-2069 1
Sga ATCCBAA-2069 2
Sga UCN34 2
Sga ATCC43143 2
Ssa SK49
Wci FBL5
Sdy ATCC12394 2
Sdy GGS124 2
Sdy AC-2713 2
Sdy RE378 2
Spy MGAS15252 1
Spy MGAS1882 1
Spy NZ131 1
Spy MGAS10270 1
Spy MGAS6180 2
Spy A20 1
Spy M1GAS 1
Spy MGAS5005 1
Sps LQ940-04
Eit DSM15952
Ehi ATCC9790
Emu QU25 DNA
Efa 1141733
Eph ATCCBAA-412
Efa D32 1
Efa OG1RF 1
Lmo J2-031
Lmo 10403S 3
Lmo SLCC5850 3
Lmo EGD
Lmo J0161 4
Lmo SLCC2482 3
Lmo R2-502
Lmo N1-011A
Lmo SLCC2540 3
Lan KCTC3501
Cmi DSM15897
Kvi DSM20405
Bca FSLF6-1037
Bth DSM20171
Fma ATCC29328
Fma SY403409CC001050417
Hsu DSM17243
Lce DSM22408
Smo F0204
Vat ACS-134-V-Col7a
Vpa ATCC17745
Vsp 6 1 27
Vsp 780strF0422
Msp UPII135-E
Ain RyC-MR95
Asp D21
Ssp CAG:314
Fal ATCC35896
Fnu 3 1 36A2
Fus 1 1 41FAA
Fus 3 1 36A2
Gbe ATCC700627
Gha M341
Tde ATCC33521
Tde ATCC35405
Tpu OMZ758
Lin 11V1-d
Pdu ATCCBAA-1640
Pan CAG:621
Eyu ATCC43715
Fba M10-2
Rla ATCC29176
Era ATCC29099
Lba NK4A179
Pal ATCC23263
Dlo AGR2136
Rsp CAG:197
Cgl PW2
Oul DSM7084
Gpa 7-10-1-b
Esp YY7918
Bbi S17
Oki DSM 17330
Lge KCTC3527
Ffr KCTC3544
Lfa KCTC3681
Lru ATCC25644
Lga JV-V03
Lho CRBIP24.179
Ljo DPC6026
Lje 27-2-CHN
Lsa UCC118
Pac D3
Plo NGRI0510Q
Wha FBL4
Lbr ATCC27305
Lbu NRRLB-30929
Lsa TMW1.1304
Lca Lc10
Lpa 8700
Lrh GG
Lfe ATCC14931
Lcu CRL705
Lco KCTC3167
Lpe KCA1
Lpl ZJ316
Sps ED99
Cla DSM 14151
Pan DSM14505
Mca PG14
Mca UFG4
Mcy C142
Mga str. F
Msy 53
Mar HAZ145 1
Mov SC01
Mmo 163K
Vsp SK1
Edo DSM3991
Csp ZWU0011
Bpe CAG:437
Ere ATCC33656
Bfi 16/4
Bsp AC2005
Eve ATCC27560
Csp CAG:230
Efa TX0012
Efa TX0012 2
Gsa NBRC15551
Nam LUREC
Fho CCUG36813
Cma M35/04/3
Sga ATCC43143 1
Sga UCN34 1
Sma ACA-DC198 1
Spa ATCC43144 1
Ssu D9
Ssu ST3 2
Ssu YB51
Spa F0449
Ssp I-G2 3
Sgo CH1 2
San C1051 4
Sin B196 1
Ssa JIM8777 3
Sth DGCC7710 1
Sth CNRZ1066 1
Sth ND03 1
Sth DGCC7710 3
Sth JIM8232 1
Sth LMD-9 5
Sth MN-ZLW-002 1

Figure S4

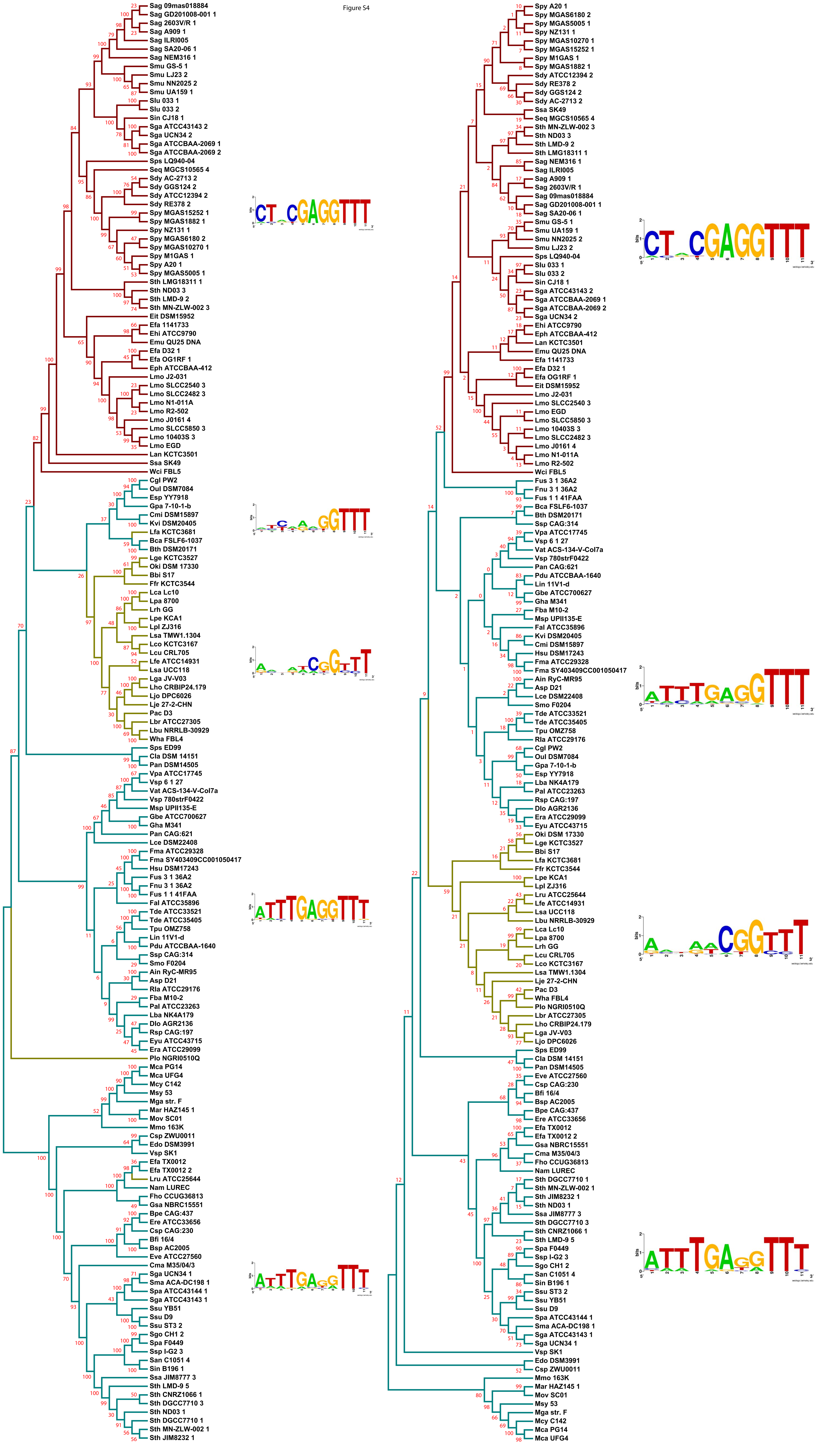
Sag 09mas018884
Sag GD201008-001 1
Sag 2603V/R 1
Sag A909 1
Sag ILRI005
Sag SA20-06 1
Sag NEM316 1
Smu GS-5 1
Smu LJ23 2
Smu NN2025 2
Smu UA159 1
Slu 033 1
Slu 033 2
Sin CJ18 1
Sga ATCC43143 2
Sga UCN34 2
Sga ATCCBAA-2069 1
Sga ATCCBAA-2069 2
Sps LQ940-04
Seq MGCS10565 4
Sdy AC-2713 2
Sdy GGS124 2
Sdy ATCC12394 2
Sdy RE378 2
Spy MGAS15252 1
Spy MGAS1882 1
Spy NZ131 1
Spy MGAS6180 2
Spy MGAS10270 1
Spy M1GAS 1
Spy A20 1
Spy MGAS5005 1
Sth LMG18311 1
Sth ND03 3
Sth LMD-9 2
Sth MN-ZLW-002 3
Eit DSM15952
Efa 1141733
Ehi ATCC9790
Emu QU25 DNA
Efa D32 1
Efa OG1RF 1
Eph ATCCBAA-412
Lmo J2-031
Lmo SLCC2540 3
Lmo SLCC2482 3
Lmo N1-011A
Lmo R2-502
Lmo J0161 4
Lmo SLCC5850 3
Lmo 10403S 3
Lmo EGD
Lan KCTC3501
Ssa SK49
Wci FBL5
Cgl PW2
Oul DSM7084
Esp YY7918
Gpa 7-10-1-b
Cmi DSM15897
Kvi DSM20405
Lfa KCTC3681
Bca FSLF6-1037
Bth DSM20171
Lge KCTC3527
Oki DSM 17330
Bbi S17
Ffr KCTC3544
Lca Lc10
Lpa 8700
Lrh GG
Lpe KCA1
Lpl ZJ316
Lsa TMW1.1304
Lco KCTC3167
Lcu CRL705
Lfe ATCC14931
Lsa UCC118
Lga JV-V03
Lho CRBIP24.179
Ljo DPC6026
Lje 27-2-CHN
Pac D3
Lbr ATCC27305
Lbu NRRLB-30929
Wha FBL4
Sps ED99
Cla DSM 14151
Pan DSM14505
Vpa ATCC17745
Vsp 6 1 27
Vat ACS-134-V-Col7a
Vsp 780strF0422
Msp UPII135-E
Gbe ATCC700627
Gha M341
Pan CAG:621
Lce DSM22408
Fma ATCC29328
Fma SY403409CC001050417
Hsu DSM17243
Fus 3 1 36A2
Fnu 3 1 36A2
Fus 1 1 41FAA
Fal ATCC35896
Tde ATCC33521
Tde ATCC35405
Tpu OMZ758
Lin 11V1-d
Pdu ATCCBAA-1640
Ssp CAG:314
Smo F0204
Ain RyC-MR95
Asp D21
Rla ATCC29176
Fba M10-2
Pal ATCC23263
Lba NK4A179
Dlo AGR2136
Rsp CAG:197
Eyu ATCC43715
Era ATCC29099
Plo NGRI0510Q
Mca PG14
Mca UFG4
Mcy C142
Msy 53
Mga str. F
Mar HAZ145 1
Mov SC01
Mmo 163K
Csp ZWU0011
Edo DSM3991
Vsp SK1
Efa TX0012
Efa TX0012 2
Lru ATCC25644
Nam LUREC
Fho CCUG36813
Gsa NBRC15551
Bpe CAG:437
Ere ATCC33656
Csp CAG:230
Bfi 16/4
Bsp AC2005
Eve ATCC27560
Cma M35/04/3
Sga UCN34 1
Sma ACA-DC198 1
Spa ATCC43144 1
Sga ATCC43143 1
Ssu YB51
Ssu D9
Ssu ST3 2
Sgo CH1 2
Spa F0449
Ssp I-G2 3
San C1051 4
Sin B196 1
Ssa JIM8777 3
Sth LMD-9 5
Sth CNRZ1066 1
Sth DGCC7710 3
Sth ND03 1
Sth DGCC7710 1
Sth MN-ZLW-002 1
Sth JIM8232 1
Spy A20 1
Spy MGAS6180 2
Spy MGAS5005 1
Spy NZ131 1
Spy MGAS10270 1
Spy MGAS15252 1
Spy M1GAS 1
Spy MGAS1882 1
Sdy ATCC12394 2
Sdy RE378 2
Sdy GGS124 2
Sdy AC-2713 2
Ssa SK49
Seq MGCS10565 4
Sth MN-ZLW-002 3
Sth ND03 3
Sth LMD-9 2
Sth LMG18311 1
Sag NEM316 1
Sag ILRI005
Sag A909 1
Sag 2603V/R 1
Sag 09mas018884
Sag GD201008-001 1
Sag SA20-06 1
Smu GS-5 1
Smu UA159 1
Smu NN2025 2
Smu LJ23 2
Sps LQ940-04
Slu 033 1
Slu 033 2
Sin CJ18 1
Sga ATCC43143 2
Sga ATCCBAA-2069 1
Sga ATCCBAA-2069 2
Sga UCN34 2
Ehi ATCC9790
Eph ATCCBAA-412
Lan KCTC3501
Emu QU25 DNA
Efa 1141733
Efa D32 1
Efa OG1RF 1
Eit DSM15952
Lmo J2-031
Lmo SLCC2540 3
Lmo EGD
Lmo SLCC5850 3
Lmo 10403S 3
Lmo SLCC2482 3
Lmo J0161 4
Lmo N1-011A
Lmo R2-502
Wci FBL5
Fus 3 1 36A2
Fnu 3 1 36A2
Fus 1 1 41FAA
Bca FSLF6-1037
Bth DSM20171
Ssp CAG:314
Vpa ATCC17745
Vsp 6 1 27
Vat ACS-134-V-Col7a
Vsp 780strF0422
Pan CAG:621
Pdu ATCCBAA-1640
Lin 11V1-d
Gbe ATCC700627
Gha M341
Fba M10-2
Msp UPII135-E
Fal ATCC35896
Kvi DSM20405
Cmi DSM15897
Hsu DSM17243
Fma ATCC29328
Fma SY403409CC001050417
Ain RyC-MR95
Asp D21
Lce DSM22408
Smo F0204
Tde ATCC33521
Tde ATCC35405
Tpu OMZ758
Rla ATCC29176
Cgl PW2
Oul DSM7084
Gpa 7-10-1-b
Esp YY7918
Lba NK4A179
Pal ATCC23263
Rsp CAG:197
Dlo AGR2136
Era ATCC29099
Eyu ATCC43715
Oki DSM 17330
Lge KCTC3527
Bbi S17
Lfa KCTC3681
Ffr KCTC3544
Lpe KCA1
Lpl ZJ316
Lru ATCC25644
Lfe ATCC14931
Lsa UCC118
Lbu NRRLB-30929
Lca Lc10
Lpa 8700
Lrh GG
Lcu CRL705
Lco KCTC3167
Lsa TMW1.1304
Lje 27-2-CHN
Pac D3
Wha FBL4
Plo NGRI0510Q
Lbr ATCC27305
Lho CRBIP24.179
Lga JV-V03
Ljo DPC6026
Sps ED99
Cla DSM 14151
Pan DSM14505
Eve ATCC27560
Csp CAG:230
Bfi 16/4
Bsp AC2005
Bpe CAG:437
Ere ATCC33656
Efa TX0012
Efa TX0012 2
Gsa NBRC15551
Cma M35/04/3
Fho CCUG36813
Nam LUREC
Sth DGCC7710 1
Sth MN-ZLW-002 1
Sth JIM8232 1
Sth ND03 1
Ssa JIM8777 3
Sth DGCC7710 3
Sth CNRZ1066 1
Sth LMD-9 5
Spa F0449
Ssp I-G2 3
Sgo CH1 2
San C1051 4
Sin B196 1
Ssu ST3 2
Ssu YB51
Ssu D9
Spa ATCC43144 1
Sma ACA-DC198 1
Sga ATCC43143 1
Sga UCN34 1
Vsp SK1
Edo DSM3991
Csp ZWU0011
Mmo 163K
Mar HAZ145 1
Mov SC01
Msy 53
Mga str. F
Mcy C142
Mca PG14
Mca UFG4

Figure S5

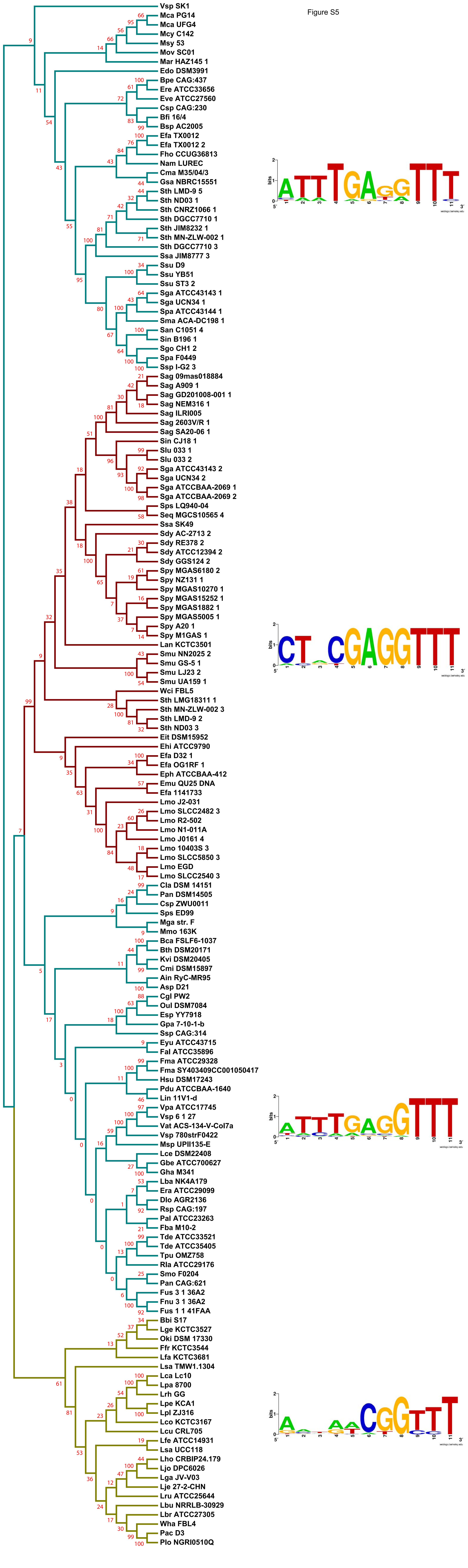

Vsp SK1
Mca PG14
Mca UFG4
Mcy C142
Msy 53
Mov SC01
Mar HAZ145 1
Edo DSM3991
Bpe CAG:437
Ere ATCC33656
Eve ATCC27560
Csp CAG:230
Bfi 16/4
Bsp AC2005
Efa TX0012
Efa TX0012 2
Fho CCUG36813
Nam LUREC
Cma M35/04/3
Gsa NBRC15551
Sth LMD-9 5
Sth ND03 1
Sth CNRZ1066 1
Sth DGCC7710 1
Sth JIM8232 1
Sth MN-ZLW-002 1
Sth DGCC7710 3
Ssa JIM8777 3
Ssu D9
Ssu YB51
Ssu ST3 2
Sga ATCC43143 1
Sga UCN34 1
Spa ATCC43144 1
Sma ACA-DC198 1
San C1051 4
Sin B196 1
Sgo CH1 2
Spa F0449
Ssp I-G2 3
Sag 09mas018884
Sag A909 1
Sag GD201008-001 1
Sag NEM316 1
Sag ILRI005
Sag 2603V/R 1
Sag SA20-06 1
Sin CJ18 1
Slu 033 1
Slu 033 2
Sga ATCC43143 2
Sga UCN34 2
Sga ATCCBAA-2069 1
Sga ATCCBAA-2069 2
Sps LQ940-04
Seq MGCS10565 4
Ssa SK49
Sdy AC-2713 2
Sdy RE378 2
Sdy ATCC12394 2
Sdy GGS124 2
Spy MGAS6180 2
Spy NZ131 1
Spy MGAS10270 1
Spy MGAS15252 1
Spy MGAS1882 1
Spy MGAS5005 1
Spy A20 1
Spy M1GAS 1
Lan KCTC3501
Smu NN2025 2
Smu GS-5 1
Smu LJ23 2
Smu UA159 1
Wci FBL5
Sth LMG18311 1
Sth MN-ZLW-002 3
Sth LMD-9 2
Sth ND03 3
Eit DSM15952
Ehi ATCC9790
Efa D32 1
Efa OG1RF 1
Eph ATCCBAA-412
Emu QU25 DNA
Efa 1141733
Lmo J2-031
Lmo SLCC2482 3
Lmo R2-502
Lmo N1-011A
Lmo J0161 4
Lmo 10403S 3
Lmo SLCC5850 3
Lmo EGD
Lmo SLCC2540 3
Cla DSM 14151
Pan DSM14505
Csp ZWU0011
Sps ED99
Mga str. F
Mmo 163K
Bca FSLF6-1037
Bth DSM20171
Kvi DSM20405
Cmi DSM15897
Ain RyC-MR95
Asp D21
Cgl PW2
Oul DSM7084
Esp YY7918
Gpa 7-10-1-b
Ssp CAG:314
Eyu ATCC43715
Fal ATCC35896
Fma ATCC29328
Fma SY403409CC001050417
Hsu DSM17243
Pdu ATCCBAA-1640
Lin 11V1-d
Vpa ATCC17745
Vsp 6 1 27
Vat ACS-134-V-Col7a
Vsp 780strF0422
Msp UPII135-E
Lce DSM22408
Gbe ATCC700627
Gha M341
Lba NK4A179
Era ATCC29099
Dlo AGR2136
Rsp CAG:197
Pal ATCC23263
Fba M10-2
Tde ATCC33521
Tde ATCC35405
Tpu OMZ758
Rla ATCC29176
Smo F0204
Pan CAG:621
Fus 3 1 36A2
Fnu 3 1 36A2
Fus 1 1 41FAA
Bbi S17
Lge KCTC3527
Oki DSM 17330
Ffr KCTC3544
Lfa KCTC3681
Lsa TMW1.1304
Lca Lc10
Lpa 8700
Lrh GG
Lpe KCA1
Lpl ZJ316
Lco KCTC3167
Lcu CRL705
Lfe ATCC14931
Lsa UCC118
Lho CRBIP24.179
Ljo DPC6026
Lga JV-V03
Lje 27-2-CHN
Lru ATCC25644
Lbu NRRLB-30929
Lbr ATCC27305
Wha FBL4
Pac D3
Plo NGRI0510Q
bits
5'
3'

Table S1

Strain	Abbreviation	Nucleotide Accession Number
Acidaminococcus intestini RyC-MR95	Ain_RyC-MR95	NC_016077
Acidaminococcus sp. D21	Asp_D21	NZ_ACGB01000044
Bifidobacterium bifidum S17	Bbi_S17	NC_014616
Brochothrix campestris FSL F6-1037	Bca_FSLF6-1037	NZ_AODH01000013
Butyrivibrio fibrisolvens 16/4	Bfi_16/4	FP929036
Bacteroides pectinophilus CAG:437	Bpe_CAG:437	FR894965
Butyrivibrio sp. AC2005	Bsp_AC2005	NZ_AUJI01000018
Brochothrix thermosphacta DSM 20171	Bth_DSM20171	NZ_JHZM01000008
Coriobacterium glomerans PW2	Cgl_PW2	NC_015389
Caryophanon latum DSM 14151	Cla_DSM_14151	NZ_MATO01000048
Catellicoccus marimammalium M35/04/3	Cma_M35/04/3	NZ_AMYT01000017
Catenibacterium mitsuokai DSM 15897	Cmi_DSM15897	NZ_ACCK01000419
Clostridium sp. CAG:230	Csp_CAG:230	FR881840
Carnobacterium sp. ZWU0011	Csp_ZWU0011	NZ_JTLM01000044
Dorea longicatena AGR2136	Dlo_AGR2136	NZ_AUJS01000026
Eubacterium dolichum DSM 3991	Edo_DSM3991	NZ_DS483477
Enterococcus faecium 1,141,733	Efa_1141733	NZ_GG688466.1
*Enterococcus_faecalis*_D32_1	Efa_D32_1	NC_018221
*Enterococcus_faecalis*_OG1RF_1	Efa_OG1RF_1	NC_017316
Enterococcus faecalis TX0012	Efa_TX0012	NZ_GL456502
Enterococcus faecalis TX0012_2	Efa_TX0012_2	NZ_GL456502
Enterococcus hirae ATCC 9790	Ehi_ATCC9790	NZ_KB946228.1
Enterococcus italicus DSM 15952	Eit_DSM15952	NZ_ALJM01000004.1
Enterococcus mundtii QU 25 DNA	Emu_QU25_DNA	NC_022878
Enterococcus phoeniculicola ATCC BAA-412	Eph_ATCCBAA-412	NZ_ASWE01000001
Eubacterium ramulus atcc 29099	Era_ATCC29099	NZ_KI271077
Eubacterium rectale ATCC 33656	Ere_ATCC33656	NC_012781
Eggerthella sp. YY7918	Esp_YY7918	NC_015738
Eubacterium ventriosum ATCC 27560	Eve_ATCC27560	NZ_DS264278.1
Eubacterium yurii subsp. margaretiae ATCC 43715	Eyu_ATCC43715	AEES01000067
Filifactor alocis ATCC 35896	Fal_ATCC35896	NC_016630
Firmicutes bacterium M10-2	Fba_M10-2	NZ_KE159700
Fructobacillus fructosus KCTC 3544	Ffr_KCTC3544	NZ_AEOP01000035.1
Facklamia hominis CCUG 36813	Fho_CCUG36813	AGZD01000007
Finegoldia magna ATCC 29328	Fma_ATCC29328	NC_010376
Finegoldia magna SY403409CC001050417	Fma_SY403409CC001050417	NZ_AFUI01000017
Fusobacterium nucleatum subsp. vincentii 3_1_36A2	Fnu_3_1_36A2	NC_022196
Fusobacterium sp. 1_1_41FAA	Fus_1_1_41FAA	NZ_GG770381.1
Fusobacterium sp. 3_1_36A2	Fus_3_1_36A2	NC_022196.1
*Gemella_bergeri*_ATCC_700627	Gbe_ATCC700627	NZ_KI271806
*Gemella_haemolysans*_M341	Gha_M341	GL883582

Gordonibacter pamelaeae 7-10-1-b	Gpa_7-10-1-b	NC_021021
Globicatella sanguinis NBRC 15551	Gsa_NBRC15551	NZ_BCQX01000009
Helcococcus sueciensis DSM 17243	Hsu_DSM17243	NZ_AUHK01000002
Kandleria vitulina DSM 20405	Kvi_DSM20405	NZ_KL370857
Lactobacillus animalis KCTC 3501	Lan_KCTC3501	NZ_GL573157.1
Lachnospiraceae bacterium NK4A179	Lba_NK4A179	NZ_ATWC01000025
Lactobacillus brevis subsp. gravesensis ATCC 27305	Lbr_ATCC27305	NZ_GG669606.1
Lactobacillus buchneri NRRL B-30929	Lbu_NRRLB-30929	CP002652
Lactobacillus casei Lc-10	Lca_Lc10	NZ_AFYT01000017.1
Lactobacillus ceti DSM 22408	Lce_DSM22408	NZ_KE383993
Lactobacillus coryniformis subsp. coryniformis KCTC 3167	Lco_KCTC3167	NZ_GL544592.1
Lactobacillus curvatus CRL 705	Lcu_CRL705	NZ_AGBU01000042.1
Lactobacillus farciminis KCTC 3681	Lfa_KCTC3681	NZ_GL575017
Lactobacillus fermentum ATCC 14931	Lfe_ATCC14931	NZ_GG669901.1
Lactobacillus gasseri JV-V03	Lga_JV-V03	NZ_GL379580
Leuconostoc gelidum KCTC 3527	Lge_KCTC3527	NZ_AEMI01000021.1
Lactobacillus hominis CRBIP 24.179	Lho_CRBIP24.179	NZ_CAKE01000018.1
Lactobacillus iners LactinV 11V1-d	Lin_11V1-d	NZ_AEHN01000016.1
Lactobacillus jensenii 27-2-CHN	Lje_27-2-CHN	NZ_KI411428.1
Lactobacillus johnsonii DPC 6026	Ljo_DPC6026	NC_017477.1
Listeria monocytogenes 10403S_3	Lmo_10403S_3	NC_018586
Listeria monocytogenes EGD	Lmo_EGD	NC_017544
Listeria monocytogenes J0161_4	Lmo_J0161_4	NC_018591
Listeria monocytogenes strain J2-031	Lmo_J2-031	NC_022568
Listeria monocytogenes strain N1-011A	Lmo_N1-011A	NC_017545
Listeria monocytogenes strain R2-502	Lmo_R2-502	NC_021837
Listeria monocytogenes serotype 7 str. SLCC2482_3	Lmo_SLCC2482_3	NC_021826
Listeria monocytogenes SLCC2540_3	Lmo_SLCC2540_3	NC_021838
Listeria monocytogenes SLCC5850_3	Lmo_SLCC5850_3	NC_018592
Lactobacillus paracasei subsp. paracasei 8700	Lpa_8700	NC_022112
Lactobacillus pentosus KCA1	Lpe_KCA1	NZ_CM001538.1
Lactobacillus plantarum ZJ316	Lpl_ZJ316	NC_020229
Lactobacillus rhamnosus GG	Lrh_GG	NC_017482
Lactobacillus ruminis ATCC 25644	Lru_ATCC25644	NZ_AFYE01000073.1
Lactobacillus sanfranciscensis TMW 1.1304	Lsa_TMW1.1304	NC_015978.1
Lactobacillus salivarius UCC118	Lsa_UCC118	NC_007929
Mycoplasma arginini HAZ145_1	Mar_HAZ145_1	NZ_AP014657.1
Mycoplasma canis PG 14	Mca_PG14	NZ_AJFQ01000005
Mycoplasma canis UFG4	Mca_UFG4	NZ_AJFU01000005.1
Mycoplasma cynos C142	Mcy_C142	NC_019949.1
Mycoplasma gallisepticum str. F	Mga_str._F	NC_017503.1
Mycoplasma mobile 163K	Mmo_163K	NC_006908
Mycoplasma ovipneumoniae SC01	Mov_SC01	NZ_AFHO01000003

Megasphaera sp. UPII 135-E	Msp_UPII135-E	NZ_AFUG01000024.1
Mycoplasma synoviae 53	Msy_53	NC_007294
Nosocomiicoccus ampullae strain LUREC	Nam_LUREC	NZ_MBFG01000013
Oenococcus kitaharae DSM 17330	Oki_DSM_17330	NZ_CM001398
Olsenella uli DSM 7084	Oul_DSM7084	NC_014363
Pediococcus acidilactici D3	Pac_D3	NZ_KB889550
Pseudoramibacter alactolyticus ATCC 23263	Pal_ATCC23263	NZ_GL622359
Peptostreptococcus anaerobius CAG:621	Pan_CAG:621	NZ_CP016534.1
Planococcus antarcticus DSM 14505	Pan_DSM14505	CAYH010000043
Peptoniphilus duerdenii ATCC BAA-1640	Pdu_ATCCBAA-1640	NZ_GL397071
Pediococcus lolii NGRI 0510Q	Plo_NGRI0510Q	NZ_BANK01000034.1
Ruminococcus lactaris ATCC 29176	Rla_ATCC29176	NZ_DS990175
Roseburia sp. CAG:197	Rsp_CAG:197	HF999864
Streptococcus agalactiae 09mas018884	Sag_09mas018884	NC_021485
*Streptococcus agalactiae*_2603V/R_1	Sag_2603V/R_1	NC_004116
*Streptococcus agalactiae*_A909_1	Sag_A909_1	NC_007432
*Streptococcus agalactiae*_GD201008-001_1	Sag_GD201008-001_1	NC_018646
Streptococcus agalactiae ILRI005	Sag_ILRI005	NC_021486
*Streptococcus agalactiae*_NEM316_1	Sag_NEM316_1	NC_004368
*Streptococcus agalactiae*_SA20-06_1	Sag_SA20-06_1	NC_019048
*Streptococcus anginosus*_C1051_4	San_C1051_4	NC_022244
*Streptococcus dysgalactiae*_subsp.equisimilis_ATCC_12394_2	Sdy_ATCC12394_2	NC_017567
*Streptococcus dysgalactiae*_subsp.equisimilis_GGS_124_1_2	Sdy_GGS124_2	NC_012891
*Streptococcus dysgalactiae*_subsp.equisimilis_AC-2713_2	Sdy_AC-2713_2	NC_019042
*Streptococcus dysgalactiae*_subsp.equisimilis_RE378_2	Sdy_RE378_2	NC_018712
*Streptococcus equi*_subsp.zooepidemicus_str.MGCS10565_4	Seq_MGCS10565_4	NC_011134
*Streptococcus gallolyticus*_subsp.gallolyticus_ATCC_43143_1	Sga_ATCC43143_1	NC_017576
*Streptococcus gallolyticus*_subsp.gallolyticus_ATCC_43143_2	Sga_ATCC43143_2	NC_017576
*Streptococcus gallolyticus*_subsp.gallolyticus_ATCC_BAA-2069_1	Sga_ATCCBAA-2069_1	NC_015215
*Streptococcus gallolyticus*_subsp.gallolyticus_ATCC_BAA-2069_2	Sga_ATCCBAA-2069_2	NC_015215
*Streptococcus gallolyticus*_UCN34_1	Sga_UCN34_1	NC_013798
*Streptococcus gallolyticus*_UCN34_2	Sga_UCN34_2	NC_013798
*Streptococcus gordonii*_str.Challis_substr.CH1_2	Sgo_CH1_2	NC_009785

*Streptococcus intermedius*_B196_1	Sin_B196_1	NC_022246
*Streptococcus infantarius*_subsp.infantarius_CJ18_1	Sin_CJ18_1	NC_016826
*Streptococcus lutetiensis*_033_1	Slu_033_1	NC_021900
*Streptococcus lutetiensis*_033_2	Slu_033_2	NC_021900
*Streptococcus macedonicus*_ACA-DC_198_1	Sma_ACA-DC198_1	NC_016749
Solobacterium moorei F0204	Smo_F0204	GL637674
*Streptococcus mutans*_GS-5_1	Smu_GS-5_1	NC_018089
*Streptococcus mutans*_LJ23_2	Smu_LJ23_2	NC_017768
*Streptococcus mutans*_NN2025_2	Smu_NN2025_2	NC_013928
*Streptococcus mutans*_UA159_1	Smu_UA159_1	NC_004350
*Streptococcus pasteurianus*_ATCC_43144_1	Spa_ATCC43144_1	NC_015600
Streptococcus parasanguinis F0449	Spa_F0449	NZ_AJMV01000063.1
Staphylococcus pseudintermedius ED99	Sps_ED99	NC_017568
Streptococcus pseudoporcinus LQ 940-04	Sps_LQ940-04	NZ_AEUY02000005.1
*Streptococcus pyogenes*_A20_1	Spy_A20_1	NC_018936
*Streptococcus pyogenes*_M1_GAS_1	Spy_M1GAS_1	NC_002737
*Streptococcus pyogenes*_MGAS10270_1	Spy_MGAS10270_1	NC_008022
*Streptococcus pyogenes*_MGAS15252_1	Spy_MGAS15252_1	NC_017040
*Streptococcus pyogenes*_MGAS1882_1	Spy_MGAS1882_1	NC_017053
*Streptococcus pyogenes*_MGAS5005_1	Spy_MGAS5005_1	NC_007297
*Streptococcus pyogenes*_MGAS6180_2	Spy_MGAS6180_2	NC_007296
*Streptococcus pyogenes*_NZ131_1	Spy_NZ131_1	NC_011375
*Streptococcus salivarius*_JIM8777_3	Ssa_JIM8777_3	NC_017595
Streptococcus sanguinis SK49	Ssa_SK49	NZ_GL890985
Subdoligranulum sp. CAG:314	Ssp_CAG:314	FR900985
Streptococcus sp.I-G2_3	Ssp_I-G2_3	NC_022584
Streptococcus suis D9	Ssu_D9	NC_017620
*Streptococcus suis*_ST3_2	Ssu_ST3_2	NC_015433
Streptococcus suis YB51	Ssu_YB51	NC_022516
*Streptococcus thermophilus*_LMG_18311_1	Sth_LMG18311_1	NC_006448
*Streptococcus thermophilus*_JIM_8232_1	Sth_JIM8232_1	NC_017581
*Streptococcus thermophilus*_CNRZ1066_1	Sth_CNRZ1066_1	NC_006449
*Streptococcus thermophilus*_DGCC7710_1	Sth_DGCC7710_1	AWVZ01000002
Streptococcus thermophilus DGCC7710_3	Sth_DGCC7710_3	AWVZ01000001
*Streptococcus thermophilus*_LMD-9_2	Sth_LMD-9_2	NC_008532
*Streptococcus thermophilus*_LMD-9_5	Sth_LMD-9_5	NC_008532
*Streptococcus thermophilus*_MN-ZLW-002_1	Sth_MN-ZLW-002_1	NC_017927
*Streptococcus thermophilus*_MN-ZLW-002_3	Sth_MN-ZLW-002_3	NC_017927
*Streptococcus thermophilus*_ND03_1	Sth_ND03_1	NC_017563
*Streptococcus thermophilus*_ND03_3	Sth_ND03_3	NC_017563
Treponema denticola ATCC 33521	Tde_ATCC33521	NZ_KB445539
Treponema denticola ATCC 35405	Tde_ATCC35405	NC_002967
Treponema putidum OMZ 758	Tpu_OMZ758	NZ_CP009228
Veillonella atypica ACS-134-V-Col7a	Vat_ACS-134-V-Col7a	NZ_AEDS01000047

Veillonella parvula ATCC 17745	Vpa_ATCC17745	NZ_ADFU01000012.1
Veillonella sp. 6_1_27	Vsp_6_1_27	NZ_GG770216.1
Veillonella sp. oral taxon 780 str. F0422	Vsp_780strF0422	NZ_AFUJ01000012.1
Virgibacillus sp. SK-1	Vsp_SK1	NZ_CCXU01000007
Weissella cibaria strain FBL5	Wci_FBL5	NZ_LVYB01000027
Weissella halotolerans FBL4	Wha_FBL4	NZ_LVVN01000018.1

Table S2

Phylum	***Class***	***Order***	***Family***	***Genus***
Firmicutes	Negativicutes	Acidaminococcales	Acidaminococcaceae	Acidaminococcus
Actinobacteria	Actinobacteria	Bifidobacteriales	Bifidobacteriaceae	Bifidobacterium
Firmicutes	Bacilli	Bacillales	Listeriaceae	Brochothrix
Firmicutes	Clostridia	Clostridiales	Lachnospiraceae	Butyrivibrio
Bacteroidetes	Bacteroidia	Bacteroidales	Bacteroidaceae	Bacteroides
Firmicutes	Bacilli	Lactobacillales	Enterococcaceae	Catellicoccus
Firmicutes	Clostridia	Clostridiales	Peptostreptococcaceae	Clostridioides
Firmicutes	Bacilli	Lactobacillales	Carnobacteriaceae	Carnobacterium
Firmicutes	Bacilli	Bacillales	Planococcaceae	Caryophanon
Actinobacteria	Coriobacteriia	Coriobacteriales	Coriobacteriaceae	Coriobacterium
Firmicutes	Erysipelotrichia	Erysipelotrichales	Erysipelotrichaceae	Catenibacterium
Firmicutes	Clostridia	Clostridiales	Lachnospiraceae	Dorea
Firmicutes	Clostridia	Clostridiales	Eubacteriaceae	Eubacterium
Firmicutes	Bacilli	Lactobacillales	Enterococcaceae	Enterococcus
Actinobacteria	Coriobacteriia	Eggerthellales	Eggerthellaceae	Eggerthella
Firmicutes				
Firmicutes	Clostridia	Clostridiales	Peptostreptococcaceae	Filifactor
Firmicutes	Bacilli	Lactobacillales	Leuconostocaceae	Fructobacillus
Firmicutes	Bacilli	Lactobacillales	Aerococcaceae	Facklamia
Firmicutes	Tissierellia	Tissierellales	Peptoniphilaceae	Finegoldia
Fusobacteria	Fusobacteriia	Fusobacteriales	Fusobacteriaceae	Fusobacterium
Firmicutes	Bacilli	Bacillales		Gemella
Actinobacteria	Coriobacteriia	Eggerthellales	Eggerthellaceae	Gordonibacter
Firmicutes	Bacilli	Lactobacillales	Aerococcaceae	Globicatella
Firmicutes	Tissierellia	Tissierellales	Peptoniphilaceae	Helcococcus
Firmicutes	Erysipelotrichia	Erysipelotrichales	Erysipelotrichaceae	Kandleria
Firmicutes	Bacilli	Lactobacillales	Lactobacillaceae	Lactobacillus
Firmicutes	Clostridia	Clostridiales	Lachnospiraceae	
Firmicutes	Bacilli	Lactobacillales	Leuconostocaceae	Leuconostoc
Firmicutes	Bacilli	Bacillales	Listeriaceae	Listeria
Tenericutes	Mollicutes	Mycoplasmatales	Mycoplasmataceae	Mycoplasma
Firmicutes	Negativicutes	Veillonellales	Veillonellaceae	Megasphaera
Firmicutes	Bacilli	Bacillales	Staphylococcaceae	Nosocomiicoccus
Firmicutes	Bacilli	Lactobacillales	Leuconostocaceae	Oenococcus

Actinobacteria	Coriobacteriia	Coriobacteriales	Atopobiaceae	Olsenella
Firmicutes	Tissierellia	Tissierellales	Peptoniphilaceae	Peptoniphilus
Firmicutes	Bacilli	Lactobacillales	Lactobacillaceae	Pediococcus
Firmicutes	Clostridia	Clostridiales	Eubacteriaceae	Pseudoramibacter
Firmicutes	Clostridia	Clostridiales	Peptostreptococcaceae	Peptostreptococcus
Firmicutes	Bacilli	Bacillales	Planococcaceae	Planococcus
Firmicutes	Clostridia	Clostridiales	Ruminococcaceae	Ruminococcus
Firmicutes	Clostridia	Clostridiales	Lachnospiraceae	Roseburia
Firmicutes	Bacilli	Lactobacillales	Streptococcaceae	Streptococcus
Firmicutes	Erysipelotrichia	Erysipelotrichales	Erysipelotrichaceae	Solobacterium
Firmicutes	Bacilli	Bacillales	Staphylococcaceae	Staphylococcus
Firmicutes	Clostridia	Clostridiales	Ruminococcaceae	Subdoligranulum
Spirochaetes	Spirochaetia	Spirochaetales	Spirochaetaceae	Treponema
Firmicutes	Negativicutes	Veillonellales	Veillonellaceae	Veillonella
Firmicutes	Bacilli	Bacillales	Bacillaceae	Virgibacillus
Firmicutes	Bacilli	Lactobacillales	Leuconostocaceae	Weissella

Table S3

Species	CRISPRdb loci No.	CRISPRdb CRISPR repeat	length	genomic CRISPR repeat	length
Sga_ATCC_43143	NC_017576_3	GTTTTTGTACTCTCAAGATTTAAGTAACCGTAAAACA	37	GTTTTTGTACTCTCAAGATTTAAGTAACCGTAAAAC	36
Sin_CJ18	NC_016826_3	GTTTTAGAGCTGTGCTGTTTCGAATGGTTCCAAAACT	37	GTTTTAGAGCTGTGCTGTTTCGAATGGTTCCAAAAC	36
Spy_MGAS15252	NC_017040_3	GTTTTAGAGCTATGCTGTTTTGAATGGTC	29	GTTTTAGAGCTATGCTGTTTTGAATGGTCCCAAAAC	36

Table S4

Protein	Group	Average Length	Low	High
Cas1	1a	301	288	307
Cas1	1b	291	261	308
Cas1	2	289	288	291
Cas1	3	299	281	304
Cas2	1a	106	99	114
Cas2	1b	102	97	109
Cas2	2	113	102	114
Cas2	3	101	96	103
Cas9	1a	1136	765	1339
Cas9	1b	1312	752	1399
Cas9	2	1347	726	1421
Cas9	3	1307	1149	1420
Csn2	1a	324	220	352
Csn2	1b	230	215	320
Csn2	2	218	168	224
Csn2	3	220	136	234
